# Overlap between EBV‐positive diffuse large B‐cell lymphoma, NOS, in a young patient and angioimmunoblastic T‐cell lymphoma: A diagnostic pitfall

**DOI:** 10.1002/ccr3.3778

**Published:** 2021-01-13

**Authors:** Chae Hwa Kim, Taban Ghaffaripour, Yi Zhou, Jennifer R. Chapman

**Affiliations:** ^1^ Division of Hematopathology Department of Pathology and Laboratory Medicine University of Miami and Sylvester Comprehensive Cancer Center Miami FL USA

## Abstract

There may be significant histopathologic overlap between EBV‐positive DLBCL, NOS, and other diagnoses including angioimmunoblastic T‐cell lymphoma (AITL). Resolution of this differential diagnosis may be particularly challenging and require extensive investigation of clinicopathologic features.

## Graphical abstract

There may be an significant histopathologic overlap between EBV‐positive DLBCL, NOS, and other diagnoses including angioimmunoblastic T‐cell lymphoma (AITL). Resolution of this differential diagnosis may be particularly challenging and require extensive investigation of clinicopathologic features.

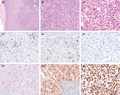

## 1 INTRODUCTION

There may be significant histopathologic overlap between EBV‐positive DLBCL, NOS, and other diagnoses including angioimmunoblastic T‐cell lymphoma (AITL). Resolution of this differential diagnosis may be particularly challenging and require extensive investigation of clinicopathologic features. We report an unusual case of cervical lymphadenopathy and retroperitoneal mass in a 34‐year‐old male patient. This case demonstrates significant histopathologic overlap between EBV‐positive diffuse large B‐cell lymphoma, NOS, and AITL, which can be a diagnostic pitfall.

## 2 PRESENTATION OF CASE

### 2.1 Clinical history

**FIGURE 1:**
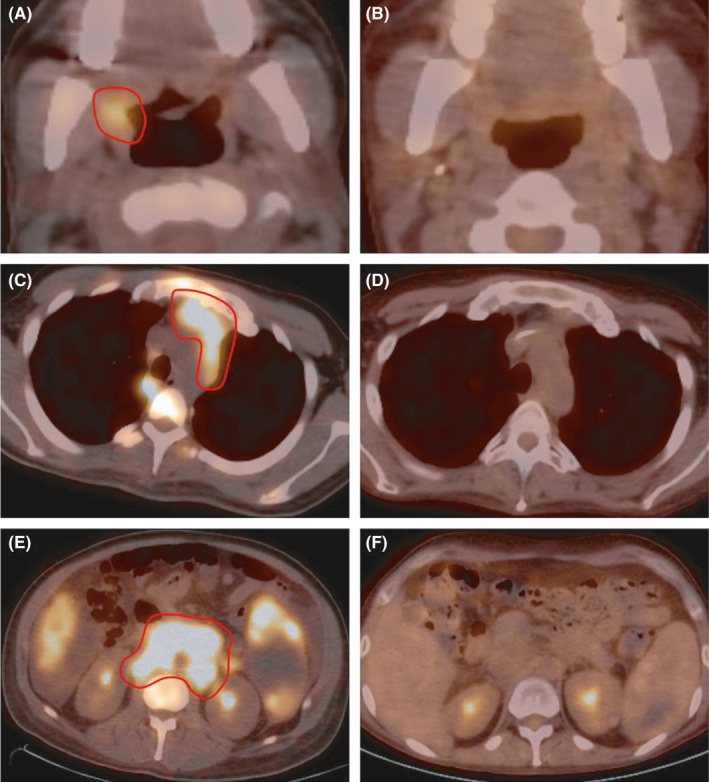
Computed tomography scan showed bilateral cervical lymphadenopathy (A) extending to the anterior mediastinum (C) and a retroperitoneal mass involving the pancreas and large vessels extending to the aortic bifurcation, as well as splenomegaly (E). Follow‐up PET‐CT after six cycles of chemotherapy demonstrated significant decrease in size of cervical lymphadenopathy (B), anterior and superior mediastinum mass (D), and upper retroperitoneal mass (F). (Red outline marks the mass in A, C, and E.)

A 34‐year‐old man with no significant medical history presented acutely with supraclavicular lymphadenopathy, fever, night sweats, and weight loss. Laboratory studies showed increased ferritin (9006 ng/mL) and lactate dehydrogenase (700 U/L), mildly increased aminotransferases levels (79 U/L), and pancytopenia (WBC 2.0 × 10^3^/µL, hemoglobin 7.6 g/dL, platelet count 32 × 10^3^/µL). Epstein‐Barr virus PCR detected 608 515 copies per mL in whole blood. Serum immunoglobulin levels were in normal range except for decreased IgM (15.75 mg/dL). Direct antiglobulin test was positive for both IgG and complement, and warm autoantibody was detected. Computed tomography scan showed bilateral cervical lymphadenopathy extending to the anterior mediastinum and a retroperitoneal mass, as well as splenomegaly (Figure 1).

### 2.2 Histopathological analysis

**FIGURE 2:**
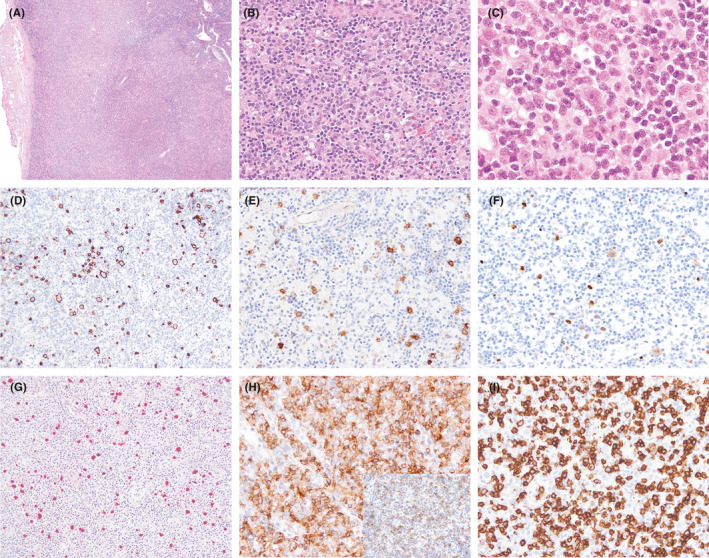
Lymph node excisional biopsy shows extensive effacement of normal nodal architecture and diffuse involvement by a polymorphic hematolymphoid infiltrate composed of small, occasional intermediate, and rare scattered large lymphoid cells (hematoxylin and eosin stain, 40× (A), 200× (B), and 500× (C) magnification). A subset of the large lymphoid cells has prominent eosinophilic nucleoli, reminiscent of those seen in Reed‐Sternberg cells and variants (C). The large lymphoid cells are positive for CD20 (D) with occasional variable coexpression of CD30 (E), and dim coexpression of PAX5 (F). EBV is detected by in situ hybridization for EBER in the majority of large lymphoid cells (G). T cells are abundant and cytologically atypical, and include both CD4 (H)‐ and CD8 (I)‐positive types. The subset of T cells shows coexpression of PD1 (inset, H). Images in panels D through IR taken at 400× magnification

**FIGURE 3:**
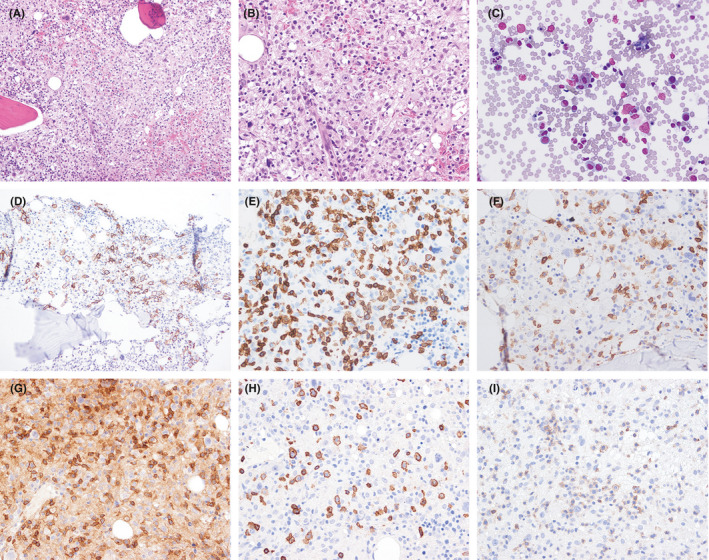
Hematoxylin and eosin–stained sections of bone marrow core biopsy show involvement by lymphoma composed of a polymorphic infiltrate of small, intermediate, and scattered large lymphoid cells (A, B, hematoxylin and eosin staining, 200× (A) and 400× (B) magnification). Bone marrow aspirate smear showing occasional hemophagocytosis (C, Wright‐Giemsa stain, 400× magnification). The large lymphoma cells expressed CD20 (D). CD3‐positive T cells are increased and cytologically atypical (E, CD3) and show diminished expression of CD7 (F, CD7). Cytologically atypical T cells include both CD4‐ and CD8‐positive types (G, CD4; H, CD8). A subset of T cells coexpress PD1 (I). Images in panels D‐I taken at 400× magnification

Histologic sections of cervical lymph node excisional biopsy showed near total nodal effacement by a hematolymphoid infiltrate causing marked paracortical expansion (Figure 2). The infiltrate was polymorphic and included scattered large atypical lymphocytes on a prominent background of abundant small‐ to intermediate‐sized lymphocytes, histiocytes, and scattered plasma cells. The large atypical cells had round or ovoid nuclei with vesicular chromatin and prominent nucleoli. A subset of the large cells had very prominent nucleoli, resembling Hodgkin and Reed‐Sternberg cells. The small‐ to intermediate‐sized background lymphoid cells also showed heterogeneity of cell size and nuclear membrane irregularities. Histologic sections of the bone marrow biopsy showed multifocal involvement by lymphoma involving approximately 30% of the sampled marrow space (Figure 3).

### 2.3 Immunohistochemistry

By immunohistochemical stains performed on the cervical lymph node biopsy, the large cells were positive for CD19, CD20 (strong), CD30 (variable intensity), CD79a (variable intensity), PAX5 (strong), MUM1, BOB1, OCT2, and BCL6 (dim, subset) while negative for CD10, CD15, and T‐cell antigens. A subset of large cells were positive for PDL1, ~30%. The background small‐ to intermediate‐sized lymphoid cells were T cells positive for CD3, CD5, and TCR‐beta, with a subset showing diminished expression of CD7. T cells were cytologically atypical and showed increased expression of PD1 and coexpression of CD10 and BCL6 in a subset (Figure 2). The cytologically atypical T cells included both CD4‐ and CD8‐positive types. CD56 or TCR‐gamma/delta‐positive T cells were rare and scattered. CD21 and CD23 highlighted dendritic networks of residual lymphoid follicles, but expanded or disrupted networks were not detected. EBER was positive in the scattered large atypical cells and in some of the intermediate‐ and small‐sized lymphoid cells. HHV‐8 was negative.

Immunophenotyping of B and T cells in the bone marrow biopsy showed patterns identical to those of the lymph node, although the T cells appeared more cytologically atypical in the bone marrow biopsy and showed more frequent loss of CD7 expression. In aspirate smears, few histiocytes demonstrating hemophagocytosis were identified.

B‐cell and T‐cell clonality analysis by multiplex polymerase chain reaction (PCR) was performed on the cervical lymph node tissue and showed a prominent and specific‐sized product peak indicating a B‐cell gene rearrangement. There was no evidence of T‐cell gene rearrangement.

## 3 DISCUSSION

The overall findings were those of polymorphic hematolymphoid infiltrate composed of EBV‐positive large B cells, including Hodgkin‐like cells, on a polymorphic but predominantly T‐cell/histiocyte‐rich background with increased and cytologically atypical T cells expressing T‐helper markers and showing diminished expression of CD7. The cytologic atypia and expansion of PD1‐positive T cells raised concern for AITL. The acute clinical presentation characterized by weight loss, fever, night sweats, lymphadenopathy, and organomegaly, positive direct antiglobulin test identifying IgG and complement, positive warm autoantibody, and suspicion for secondary HLH syndrome furthered this suspicion. However, the absence of expansion of follicular dendritic networks, presence of more cytologic atypia on CD8‐positive T cells, and absence of detectable T‐cell clonality collectively did not favor this consideration. Although an EBV‐related polymorphic lymphoproliferative lesion was also considered, the patient had no identifiable causes of immunosuppression. An interpretation of EBV^+^DLBCL, NOS, and the Ann Arbor stage III, with secondary HLH syndrome, was therefore made. International Prognostic Score (IPI) score for risk stratification was 3. EPOCH‐R chemotherapy regimen was started, and six cycles were completed resulting in partial remission. Follow‐up PET‐CT demonstrated significant decrease in size of cervical lymphadenopathy, anterior and superior mediastinum mass, upper retroperitoneal mass, and spleen (Figure 1).

Epstein‐Barr virus–positive diffuse large B‐cell lymphoma, not otherwise specified (EBV^+^ DLBCL, NOS), was formerly designated as EBV‐positive DLBCL of the elderly, but the restriction to elderly patients has been removed given that the neoplasm is now recognized to occur over a wide age range. While most studies have shown similar poor survival in all patients regardless of age, more recent reports have described a significantly better overall survival in young patients.^1^ The majority of EBV^+^ DLBCL, NOS, are composed of only scattered or loosely clustered large lymphoma cells, frequently with Hodgkin‐like features, amidst a prominent polymorphic reactive hematolymphoid background, and in these cases, the diagnosis may be more challenging due to overlapping features with other lymphomas. In cases in which scattered large B cells are present on a background rich in T cells and histiocytes, the morphologic and immunophenotypic features may raise the differential possibility of T‐cell/histiocyte‐rich large B‐cell lymphoma (TCHRLBCL).^2^ In such cases, the identification of EBV present within the large lymphoma cells would argue against the diagnosis of TCHRLBCL and point toward the possibility of an EBV‐related large B‐cell lymphoma or other T‐cell lymphoma containing EBV‐positive large B cells.

When background populations are polymorphic in appearance and include small lymphocytes, histiocytes, plasma cells, and immunoblasts, this pattern may raise the diagnostic possibilities of classic Hodgkin lymphoma (CHL), EBV‐related polymorphic hematolymphoid proliferations arising in the context of immunosuppression, or AITL. In these circumstances, differentiating these lesions from classic Hodgkin lymphoma can be done by immunophenotyping in almost all cases. History of immunosuppression is required to exclude EBV‐related polymorphic‐type proliferations. As in the case presented here, separating EBV^+^ DLBCL, NOS, from AITL, can be challenging in clinical practice, and to our knowledge, this diagnostic pitfall is not specifically reported.

Angioimmunoblastic T‐cell lymphoma is a relatively common subtype of peripheral T‐cell lymphoma of T‐helper cell origin characterized histologically by the frequent presence of a polymorphic hematolymphoid infiltrate within which the neoplastic T cells may be the minority of cells. The nearly constant association of AITL with EBV has led to the suggestion of a possible etiologic role for the virus, possibly through antigen stimulation or immune dysregulation.^3^ The features of polymorphic background, scattered large and EBV‐positive B cells, and the presence of small T cells are typical of AITL and are overlapping with those of EBV^+^DLBCL, NOS.

In patients with established AITL, high‐grade histologic progression, when occurring on infrequent occasions, is almost always to EBV^+^ DLBCL (AITL progressing to EBV^+^ DLBCL).^4^ Simultaneous presentation of EBV^+^ DLBCL and AITL in the same anatomical site (composite AITL/B‐cell lymphoma) is also described.^5^ While the pathogenesis underlying progression to DLBCL in the context of AITL has not been fully elucidated, hypothetical models proposed by Dunleavy et al suggest CXCL13 mediated co‐stimulatory loop of EBV‐infected B cells and T cells.^6^

In the case we describe, T cells were abundant, heterogeneous, and cytologically atypical in appearance, showed diminished expression of CD7, and were enriched for T cells expressing PD1, creating overlapping features with those of T‐cell lymphomas of T‐helper immunophenotype and specifically with AITL. While the presence of atypical T cells occurring in the background of nodular lymphocyte‐predominant Hodgkin lymphoma has been reported, the presence of such atypical T cells is unusual in EBV‐positive DLBCL, NOS, and to our knowledge has not been reported in the context of this lymphoma.^7^ We hypothesize that the cytologic atypia among T cells and expansion of T‐helper cells may be a reactive response possibly to the presence of the EBV‐related B‐cell lymphoma, HLH syndrome, or both. More studies are needed to elucidate the complicated relationship between EBV^+^ DLBCL and its microenvironment. In the meantime, this case highlights the importance of awareness of this potential diagnostic pitfall, careful morphologic review, comprehensive immunohistochemical studies, and integration of ancillary testing results including clonality assays in classification of these lymphomas.

## CONFLICT OF INTEREST

None declared.

## AUTHOR CONTRIBUTIONS

CHK: wrote and edited the manuscript. TG: contributed to the editing of the figures. YZ: reviewed the slides and flow cytometry. JRC: supervised and revised the manuscript.

## ETHICAL APPROVAL

All procedures performed in the study were in accordance with the ethical standards of the institutional research committee and with the 1964 Helsinki Declaration and its later amendments or comparable ethical standards. This study does not require any ethical committee approval.

## Data Availability

The authors confirm that the data supporting the findings of this study are available within the article.
